# Antibiotic resistance in potential probiotic lactic acid bacteria of fermented foods and human origin from Nigeria

**DOI:** 10.1186/s12866-023-02883-0

**Published:** 2023-05-19

**Authors:** Rachael T. Duche, Anamika Singh, Arundhati Ganesh Wandhare, Vikas Sangwan, Manvesh Kumar Sihag, Tochukwu N. T. Nwagu, Harsh Panwar, Lewis. I. Ezeogu

**Affiliations:** 1 Department of Dairy Microbiology, Guru Angad Dev Veterinary and Animal Sciences University, Ludhiana, Punjab India; 2 Department of Microbiology, Federal University of Agriculture Makurdi-Nigeria, Makurdi, Nigeria; 3 Department of Dairy Chemistry, Guru Angad Dev Veterinary and Animal Sciences University, Ludhiana, Punjab India; 4 Department of Microbiology, University of Nigeria Nsukka, Nsukka, Nigeria; 5 UNESCO International Centre for Biotechnology, Nsukka, Nigeria

**Keywords:** Antibiotic resistance, Lactic acid bacteria, Lactobacilli, Multi drug resistant, Resistance genes

## Abstract

**Introduction:**

Probiotic lactobacilli are generally recognized as safe (GRAS) and are being used in several food and pharma formulations. However, growing concern of antibiotic resistance in bacterial strains of food origin and its possible transmission via functional foods is increasingly being emphasized.

**Objectives:**

This study screened potential probiotic lactic acid bacteria (LAB) strains for their phenotypic and genotypic antibiotic resistance profiles.

**Methods:**

Susceptibility to different antibiotics was assayed by the Kirby Bauer standard disc diffusion protocol. Both conventional and SYBR-RTq-PCR were used for detection of resistance coding genes.

**Results:**

A variable susceptibility pattern was documented against different antibiotic classes. LAB strains irrespective of origin displayed marked phenotypic resistance against cephalosporins, aminoglycosides, quinolones, glycopeptides; and methicillin among beta-lactams with few exceptions. In contrast, high sensitivity was recorded against macrolides, sulphonamides and carbapenems sub-group of beta-lactams with some variations. parC, associated with ciprofloxacin resistance was detected in 76.5% of the strains. Other prevalent resistant determinants observed were aac(6?)Ii (42.1%), ermB, ermC (29.4%), and tetM (20.5%). Six (?17.6%) of the isolates were free from genetic resistance determinants screened in this study.

**Conclusion:**

Study revealed presence of antibiotic resistance determinants among lactobacilli from both fermented foods and human sources.

## Introduction

Substantial evidence led benefits of probiotics have spurred massive interest in their characterization and application for nutrition and health [1]. Having GRAS (generally recognized as safe) and QPS (qualified presumption of safety) status, LABs are often being added to foods for specific health benefits. Rising antimicrobial resistance (AMR) is substantial threat to public health and economy. Antibiotics become less effective due to ever-rising numbers of multidrug-resistant (MDR) pathogenic strains. While AMR microbial infections (global annual mortality rates of > 700,000–1 million, and projected to reach 10 million by 2050) [2] have drawn considerable scientific and medical attention, evidence has been growing of continuous gene exchange between pathogenic strains and ostensibly harmless or even beneficial commensal species. The implication is that the latter are now considered “reservoirs” of antibiotics resistance genes (ARGs), which, through multiple pathways, may propagate and eventually share with pathogens or pathobionts [3–5]. In recent times, LAB strains displaying single or multiple antibiotic resistance phenotypes have been reported; spurring concerns that these genes may, by genetic mechanisms, be acquired by human and animal pathogens [6, 7]. Already, reports have been made of conjugative LAB-to-pathogen resistance coding gene transfer [7–9], leading to the advocation by the European Food Safety Authority (EFSA) and WHO of the exclusion of bacterial strains carrying mobile genetic elements with ARGs from feeds, food fermentations, and probiotic use [10, 11]. Consequently, possession of resistance phenotypes by LAB, the location of the resistant genes, and the transmissibility of these traits have become hot topics for intensive research.

Probiotic strains are commonly isolated from traditional fermented foods and milk products. Recent research reports suggests that LAB strains from human origin (milk, infant faecal samples) may have better survival against gastric and intestinal stress factors; thereby making them better probiotic candidates [12, 13]. This study aimed at analysing potential probiotic bacteria from indigenous Nigerian fermented foods and human sources for their antibiotic resistance phenotypes and genotypes, to clarify the possibility that they may propagate antibiotic resistance genes, as well as their suitability for probiotic and food production application. Few recent studies establish strong linkage between fermented food consumption and transfer of antibiotic resistant strains to consumers [14–16]. Although the mobilization of resistance determinants among LABs, LABs to other pathogens, humans and animals was not taken up in this study, the study highlights the presence of resistance coding genes in GRAS strains and proposes future risk assessment studies for accurate estimates of the level of threat that probiotic strains may pose at food-human-animal-environment interface.

## Materials and methods

### LAB isolation and identification

Lactobacilli were isolated from local Nigerian fermented food sources [Garri (n = 10), Akpu (n = 02), Kunu (n = 05), Pito (n = 01), Burukutu (n = 03) and Fura da nono (fermented cow milk) (n = 01)]; as well as from human milk (n = 05), and healthy human infant (ages 1–24 months) faecal samples (n = 07) from Makurdi, Benue State, Nigeria. All the samples were collected in pre-sterilized glass bottles and transported to laboratory under refrigerated conditions. Fermented food samples were collected from local vendors; human milk samples were from volunteer healthy mothers attending the Bishop Murray Medical Centre, Makurdi. Before collection of milk, nipple and mammary areola were cleaned with water and 70% ethanol. Milk was obtained *via* manual expression using sterile gloves. First few milk drops were discarded. Infant human faecal samples were collected from healthy human infants with no history of medication in past 04 weeks. Fresh stool samples were collected in 0.5% L-cysteine HCL supplemented de Man Rogosa and Sharpe (Lc-MRS) tubes using sterile swabs. All volunteers or their guardians gave consent to the protocol and purpose of study. Isolation was carried out by plating appropriate serial dilutions over Lc-MRS agar (HiMedia Labs) plates, followed by incubation in an anaerobic jar with gaspack at 37^o^C for 24–48h. *Lacticaseibacillus rhamnosus* GG (LGG) ATCC 53103 was taken as reference strain in this study. Gram – positive, catalase and oxidase negative bacilli were subjected to genus-specific PCR using LbLMA1 (5’ – CTCAAAACTAAACAAAGTTTC – 3’) and R161 (5’ – CTTGTACACACCGCCCGTCA – 3’) primer pair targeting 16SrRNA region [17–19]; and MALDI-TOF-MS (Matrix Assisted Laser Desorption Ionization – Time-of-Flight Mass Spectrometry) (BioMerieux, France) for species level identification. Log scores of ≥ 1.7 were indicative of close relationships at the genus level, while score values of ≥ 2 were taken as threshold for matches at the species level. Isolates with log scores of ≥ 2 were accepted as correctly identified. MALDI-TOF-MS has emerged as a potential tool for microbial identification and has been approved for identification of cultured bacteria by FDA and other regulatory agencies [20]. Identities of shortlisted cultures were also validated by 16SrRNA gene sequencing [18]. Amplified products were sequenced using and external DNA sequencing service.

Isolates, selected based on cultural similarity, genus-specifc PCR and MALDI-TOF identity, were subjected to sequencing using 16S rRNA Forward 5’- CCAGAGTTTGATCMTGGCTCAG − 3’ and Reverse 5’- CGGTTACCTTGTTACGACTTCACC − 3’ primers [18]. Amplified products of 1400 bp were sequenced using an external DNA sequencing service (NXGenBio Life Sciences, New Delhi). Sequences were edited using BioEdit (Finch-TV version 1.4.0), and thereafter compared with sequences on the NCBI database using the BLASTn algorithm (blast.ncbi.nlm.nih.gov/Blast.cgi). The alignment was done manually with cognizance to missing nucleotides before the phylogenetic tree was constructed using sequence viewer (MEGA X software version 10.0.5).

### Antibiotic susceptibility profiling

#### Phenotypic profile

Susceptibility to 27 different antibiotics (Table 1) was assayed by the Kirby Bauer standard disc diffusion protocol as modified in Wang et al. 2022 [9]. Briefly, 200 µL inoculum (0.5 McFarland, approx. 10^8^ CFU/mL) of overnight grown culture were evenly spreaded over MRS agar plates and allowed to dry at room temperature for 5 min. Antibiotic discs (Hi-Media Labs) were placed equidistance using sterile forceps. Plates were pre-incubated at room temperature to ensure proper diffusion of antibiotics, and the zones of inhibition (ZOI) were measured using antibiotics zone scale (Hi-Media Labs) after overnight incubation at 37˚C.

**Table 1:** Antibiotics used in the study along with their class and mode of antibacterial action

S. No.	Antibiotic (Disc Code)	Standard Concentration (µg/ml)	Antimicrobial Class	Activity
1	Ciprofloxacin (CIP)	5	Quinolones	DNA Replication Inhibitors
2	Norfloxacin (NX)	10		
3	Gatifloxacin (GAT)	5		
4	Moxifloxacin (MO)	5		
5	Nalidixic acid (NA)	30		
6	Gentamycin (GEN)	10	Aminoglycosides	Inhibitors of protein synthesis
7	Tobramycin (TOB)	10		
8	Kanamycin (K)	30		
9	Clindamycin (CD)	2	Lincosamides	
10	Azithromycin (AZM)	15	Macrolides	
11	Erythromycin (E)	15		
12	Tetracycline (TE)	30	Tetracyclines	
13	Fusidic acid (FC)	10	Fusidane	
14	Tigecycline (TGC)	15	Glycylcyclines	
15	Methicillin (MET)	5	β-Lactams	Inhibitors of cell wall synthesis
16	Penicillin G (P)	10	Penicillins	
17	Amoxiclav (Amoxicillin-clavulanate) (AMC)	30		
18	Imipenem (IPM)	10	Carbapenems	
19	Meropenem (MRP)	10		
20	Vancomycin (VA)	30	Glycopeptides	
21	Teicoplanin (TEI)	30		
22	Cefoxitin (CX)	30	Cephalosporins	
23	Cefmetazole (CMZ)	30	2^nd^ generation Cephalosporins	
24	Ceftazidime (CAZ)	30	3^rd^ generation Cephalosporins	
25	Polymyxin B (PB)	300	Polymyxins	
26	Cotrimoxazole (COT)	25	Sulfonamides	Interfere with folic acid synthesis and other metabolic processes
27	Trimethoprim (TM)	10		

The antibiotic susceptibility breakpoints are best established for clinically important microorganisms. Lactobacilli displays intrinsic resistance to several antibiotics, likewise in general lactobacilli shows high level of resistance to vancomycin [21]; while *L. plantarum* and *L. pentosus* possess resistance to streptomycin. CLSI and EUCAST provide breakpoints for only couple of antibiotics testing (ampicillin, clindamycin, chloramphenicol, and erythromycin) [22], and recommends minimum inhibitory concentration (MIC) determination for antibiotic susciptiblity testing for lactobacilli. However, as the present study aimed at genotypic profiling of resistance genes, phenotypic resistotyping was carried out by standard agar disk diffusion method for determination of antibiotic susceptibility patterns following earlier published reports [23–26]. Isolates showing resistance to ≥ 3 antibiotic classes were considered multidrug resistant (MDR). Multiple antibiotic resistance (MAR) index was calculated using the Gyorgy et al. 2021 [27] method.

#### Genotypic profile

##### Detection of antibiotic resistance genes

All the strains were tested for the presence of target genes using conventional and SYBR-RTq-PCR. Gene amplification was carried out using primers amplifying determinants responsible for resistance to specific antibiotics. Target genes, respective primer pairs, amplicon sizes, and annealing temperature are presented in Table 2. PCR reaction mixtures (25 µl) contained 5 µl of reaction buffer,1 µl of purified DNA (50ng), 1 mM of each specific primer set, 0.1 mM of each dNTP (2.5 mM), and 1U of Taq polymerase (Takara Bio). RTqPCR reaction mixtures (25 µl) comprised of SYBR green master mix (12.5 µl), primer pair (1 mM each) and template DNA (50ng). Bacterial DNA templates for PCR amplifications were obtained according to Pospiech and Neumann [38]. Reaction conditions for DNA amplification consisted of an initial denaturation for 5 min at 95°C, 35 cycles each of denaturation (95°C/40s), annealing (refer Table 2) and extension (72°C/70s); followed by final extension at 72°C/20 min. Fluorescence was recorded during extension, for generation of amplification curves. For RTqPCR, melt curve and peak analysis were carried out at melting rate value of 0.2^o^C/min from 65 to 95^o^C. Target gene specific amplicons in conventional PCR and amplification curves/ specific melting peaks (RTqPCR) confirmed presence or absence of target genes.

**Table 2:** Primer pairs and PCR conditions used for detection of selected antibiotic resistance coding genes

Determining resistance to	Target gene		Primer sequence (5’→3’)		Amplicon size (bp)		Annealing temperature (°C)	Reference	
Tetracyclines	*tetM*		GTG GAC AAA GGT ACA ACG AG CGG TAA AGT TCG TCA CAC AC		406		60.5	[28]	
	*tetK*		GAT CAA TTG TAG CTT TAG GTG AAG G TTT TGT TGA TTT ACC AGG TAC CAT T		155		60.5		
	*tetL*		TGG TGG AAT GAT AGC CCA TT CAG GAA TGA CAG CAC GCT AA		229		60.5		
	*tetO*		AAC TTA GGC ATT CTG GCT CAC TCC CAC TGT TCC ATA TCG TCA		515		60.5		
	*tetW*		GAG AGC CTG CTA TAT GCC AGC GGG CGT ATC CAC AAT GTT AAC		168		60.5	[29]	
Macrolides and lincosamides	*ermA*		CCC GAA AAA TAC GCA AAA TTT CAT CCC TGT TTA CCC ATT TAT AAA CG		590		60.5	[28]	
	*ermB*		TGG TAT TCC AAA TGC GTA ATG CTG TGG TAT GGC GGG TAA GT		745		60.5		
	*mefA/E*		CAA TAT GGG CAG GGC AAG AAG CTG TTC CAA TGC TAC GC		317		60.5		
	*ermC*		AAT CGT CAA TTC CTG CAT GT TAA TCG TGG AAT ACG GGT TTG		299		60.5	[30]	
	*lnuA*		GGT GGC TGG GGG GTA GAT GTA TTA ACT GG GCT TCT TTT GAA ATA CAT GGT ATT TTT CGA TC		323		60.5	[29]	
Aminoglycosides	*aac(6’)-Ie-aph(2”)-Ia*		CAG AGC CTT GGG AAG ATG AAG CCT CGT GTA ATT CAT GTT CTG GC		348		57	[31]	
	*aph3IIIa*		GGC TAA AAT GAG AAT ATC ACC GG CTT TAA AAA ATC ATA CAG CTC GCG		523		57		
	*ant(4)-Ia*		CAA ACT GCT AAA TCG GTA GAA GCC GGA AAG TTG ACC AGA CAT TAC GAA CT		294		57		
	*aph(2”)-Ic*		CCA CAA TGA TAA TGA CTC AGT TCC C CCA CAG CTT CCG ATA GCA AGA G		444		57		
	*aph(2”)-Id*		GTG GTT TTT ACA GGA ATG CCA TC CCC TCT TCA TAC CAA TCC ATA TAA CC		641		57		
	*ant(6)-Ia*		CGG GAG AAT GGG AGA CTT TG CTG TGG CTC CAC AAT CTG AT		563		57	[32]	
	*aac(6’)-Ii*		TGG CCG GAA GAA TAT GGA GA GCA TTT GGT AAG ACA CCT ACG		410		57		
	*aadE*		ATG GAA TTA TTC CCA CCT GA TCA AAA CCC CTA TTA AAG CC		1060		51	[33]	
Penicillins	*blaZ*		ACT TCA ACA CCT GCT GCT TTC TAG GTT CAG ATT GGC CCT TAG		240		60.5	[34]	
	*mecA*		AGT TCT GCA GTA CCG GAT TTG C AAA ATC GAT GGT AAA GGT TGG C		533		57	[35]	
	*int-Tn (Tn916/* *Tn1545)*		GCG TGA TTG TAT CTC ACT GAC GCT CCT GTT GCT TCT		1028		57	[36]	
	*bla*		CAT ART TCC GAT AAT ASM GCC CGT STT TAA CTA AGT ATS GY		297		51	[34]	
Vancomycin	*vanE*		TGT GGT ATC GGA GCT GCA G GTC GAT TCT CGC TAA TCC		513		51	[29]	
Trimethoprim	*dfrA*		CTT TTC TAC GCA CTA AAT GTA AG CAT TAT CAA TAA TTG TCG CTC AC		474		51	[37]	
	*dfrD*		GGA AGG GCT TTA CCT GAC AGA AG CGA CAT AAG GCA AGA ACA TAA CAT A		175		51	[37]	
Ciprofloxacin	*parC*		TAT TCY AAA TAY ATC ATT CAR GA GCY TCN GTA TAA CGC ATM GCC G		286		51	[34]	

## Results

### Identification of isolates

**Fig. 1:**
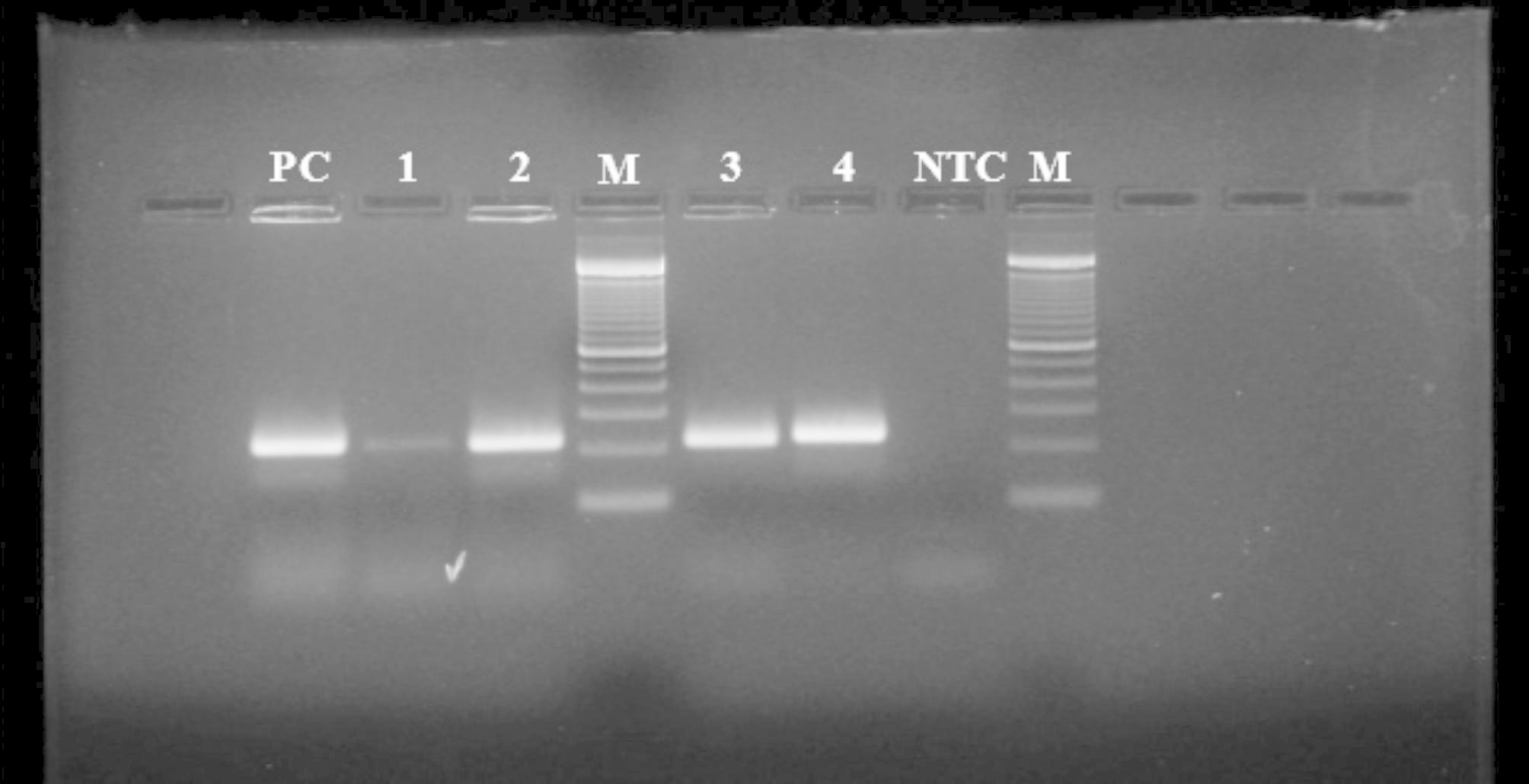
Representative agarose gel electrophoresis for genus specific PCR. NTC, No template control; Lanes 1–4, PCR product for tentative lactobacilli isolates; PC, Positive control (PCR product with *Lacticaseibacillus rhamnosus* GG DNA); M, 100 bp DNA Ladder. PCR products were electrophoresed in 1.5% agarose gel at 100 V

**Fig. 2:**
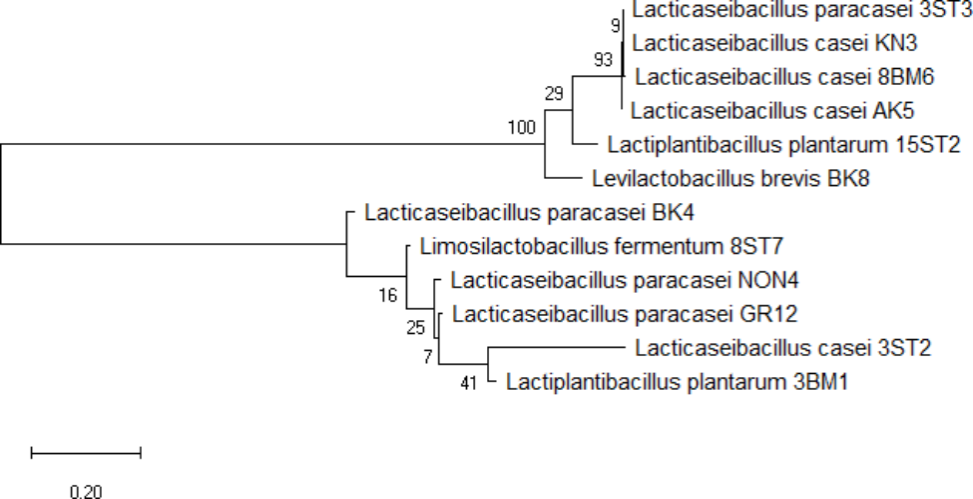
Phylogenetic tree of LAB isolates generated using neighbor-joining method in MEGA 6.0. Values shown in each node corresponds to bootstrap values

Thirty-four isolates showing Gram positive, catalase and oxidase negative reaction were subjected to genus-specific PCR. Amplicon size of ~ 250 bp confirmed lactobacilli (Fig. 1). Isolates were successfully identified to species level by MALDI-TOF MS (Table 3). Isolates from different species and different origin were randomly selected for identification and re-validation by 16 S rRNA sequencing. The MALDI-TOF MS identity matched with the 16 S rRNA sequencing outcomes for 66.7% (8/12) of the test isolates (3ST2, 3ST3, 8BM6, 15ST2, KN3, BK4, BK8, and AK5). While, 33.3% (4/12) of isolates (3BM1, GR12, NON4 and 8ST7) revealed different identity upon sequencing. While the MALDI-TOF best match identity for isolate 15ST2 was *Lactiplantibacillus pentosus*, and the second-best match *Lactiplantibacillus plantarum*; sequencing data simply identified it as *Lactiplantibacillus plantarum.* Evolutionary analyses was conducted in MEGA X software using the Maximum Likelihood Method and Tamura-Nei model with 500 bootstrap. Overall the isolates are clustered into two distinctly related clades. One clad consist of isolates 3ST3, KN3, 8BM6, AK5, 15ST2 and BK8 indicating the possible evolution of these isolates from a same common ancestor. While isolates BK4, 8ST7, NON4, GR12, 3ST2 and 3BM1 are clustered in another single clad, which might not share an immediate common ancestor among themselves. However, during a course of evolution these isolates possibly have originated from a distinctly related common ancestor. Within a clad, isolates 3ST3 (*Lacticaseibacillus paracasei*), KN3 (*Lacticaseibacillus casei*), 8BM6 (*Lacticaseibacillus casei*), and AK5 (*Lacticaseibacillus casei*) are more closely related indicating their evolution from a same common origin. Whereas, isolates BK8 (*Levilactobacillus brevis*) and isolate 15ST2 (*Lactiplantibacillus platntarum*) branched into a separate sub-clad which share a distinctly related common origin with L*acticaseibacillu*s *paracasei* and *Lacticaseibacillus casei* isolates (3ST3, KN3, 8BM6 and AK5) (Fig. 2).

**Table 3:** Identity of isolates and reference strains used in the study along with their respective origin

S. No.	Source	Origin	Strain – Lab Identity	Identity	Identification by
1	Human Source	Infant feces at 3 m	3ST2	*Lacticaseibacillus casei*	G, M, 16 S
2		Infant feces at 3 m	3ST3	*Lacticaseibacillus paracasei*	G, M, 16 S
3		Infant feces at 3 m	3ST5	*Lacticaseibacillus casei*	G, M
4		Infant feces at 3 m	3ST7	*Lacticaseibacillus casei*	G, M
5		Human milk at 3 m	3BM1	^**M**^ *Levilactobacillus brevis* ^**16S**^ *Lactiplantibacillus plantarum*	G, M, 16 S
6		Human milk at 3 m	3BM3	*Lacticaseibacillus casei*	G, M
7		Human milk at 3 m	3BM4	*Lacticaseibacillus paracasei*	G, M
8		Human milk at 8 m	8BM6	*Lacticaseibacillus casei*	G, M, 16 S
9		Human milk at 8 m	8BM9	*Levilactobacillus brevis*	G, M
10		Infant feces at 8 m	8ST5	*Lactiplantibacillus pentosus*	G, M
11		Infant feces at 8 m	8ST7	^**M**^ *Lactiplantibacillus pentosus* ^**16S**^ *Limosilactobacillus fermentum*	G, M, 16 S
12		Infant feces at 15 m	15ST2	^**M**^ *Lactiplantibacillus pentosus* ^**16S**^ *Lactiplantibacillus plantarum*	G, M, 16 S
13	Fermented Nigerian Foods	*Fura da nono*	NON4	^***M***^ *Levilactobacillus brevis* ^**16S**^ *Lacticaseibacillus paracasei*	G, M, 16 S
14		*Kunu*	KN3	*Lacticaseibacillus casei*	G, M, 16 S
15		*Kunu*	KN5	*Levilactobacillus brevis*	G, M
16		*Kunu*	KN6	*Lacticaseibacillus casei*	G, M
17		*Kunu*	KN9	*Levilactobacillus brevis*	G, M
18		*Kunu*	KN10	*Levilactobacillus brevis*	G, M
19		*Garri*	GR5	*Levilactobacillus brevis*	G, M
20		*Garri*	GR4	*Lacticaseibacillus casei*	G, M
21		*Garri*	GR11	*Lacticaseibacillus paracasei*	G, M
22		*Garri*	GR8	*Lacticaseibacillus casei*	G, M
23		*Garri*	GR12	^**M**^ *Lactiplantibacillus plantarum* ^**16S**^ *Lacticaseibacillus paracasei*	G, M, 16 S
24		*Garri*	GR13	*Levilactobacillus brevis*	G, M
25		*Garri*	GR2	*Levilactobacillus brevis*	G, M
26		*Garri*	GR27	*Lacticaseibacillus casei*	G, M
27		*Garri*	GR29	*Levilactobacillus brevis*	G, M
28		*Garri*	GR32	*Lacticaseibacillus casei*	G, M
29		*Burukutu*	BK4	*Lacticaseibacillus paracasei*	G, M, 16 S
30		*Burukutu*	BK5	*Lacticaseibacillus paracasei*	G, M
31		*Burukutu*	BK8	*Levilactobacillus brevis*	G, M, 16 S
32		*Pito*	PT1	*Lacticaseibacillus paracasei*	G, M
33		Fermenting *Akpu*	AK1	*Lacticaseibacillus casei*	G, M
34		Fermenting *Akpu*	AK5	*Lacticaseibacillus casei*	G, M, 16 S
35	RS	Reference strain	LGG -ATCC 53,103	*Lacticaseibacillus rhamnosus* GG	G, M

G, genus-specific PCR; M, MALDI-ToF; 16 S, 16 S rDNA sequencing; m, months; RS, reference strain.

### Antibiotic resistance profiles

**Fig. 3:**
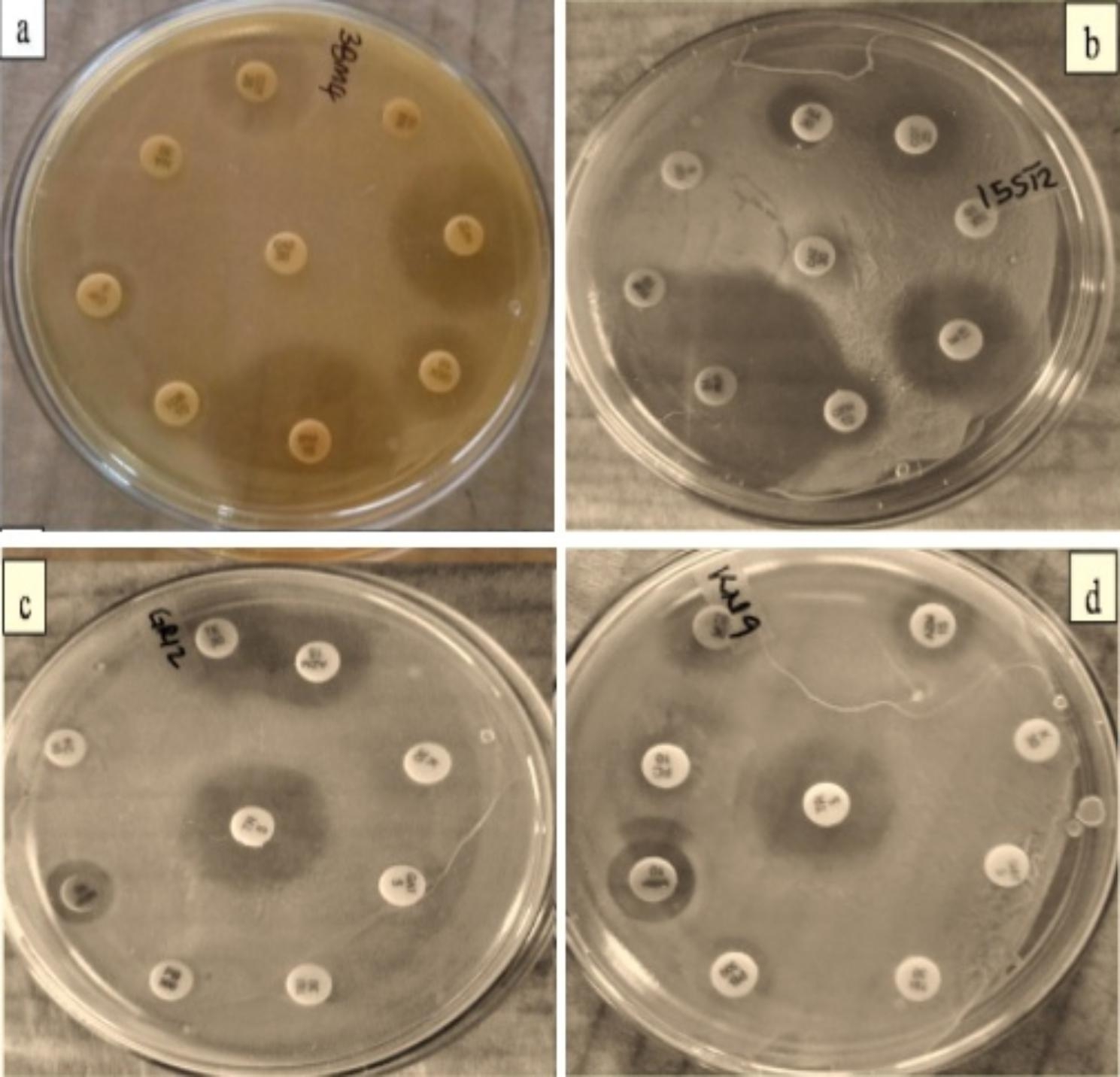
Antibiotic susceptibility pattern of representative LAB isolates against different antibiotics was determined by disk diffusion assay. (**a**) *Lacticaseibacillus paracasei* 3BM4 (**b**) *Lactiplantibacillus plantarum* 15ST2 (**c**) *Lacticaseibacillus paracasei* GR12 (**d**) *Levilactobacillus brevis* KN9

**Fig. 4:**
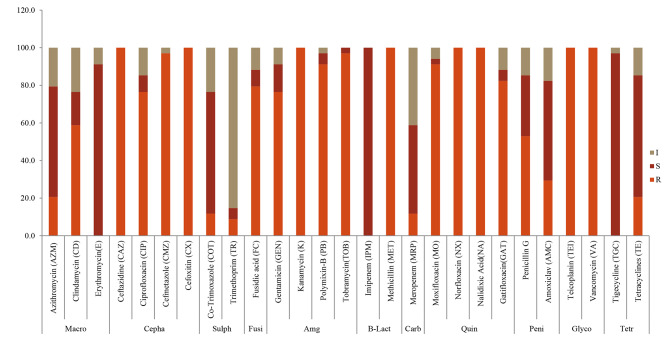
Antibiotic susceptibility pattern (%) (resistant: R; intermediate sensitive: I; and sensitive: S) displayed by lactic acid bacteria strains against different antibiotics: Macro, macrolides; Cepha, cephalosporines; Sulph, sulphonamides; Fusi, fusidanes; Amg, aminoglycosides; B-Lact, Beta-lactams; Carb, carbapenems; Quin, quinolones; Peni, penicillins; Glyco, glycopeptides; Tetr, tetracyclines

Antibiotic susceptibility was classified as resistant (R), intermediate susceptible (I) and susceptible (S), respectively, depending on microbial responses (Fig. 3 and Table 4**)**. Isolates displayed marked resistance against cephalosporins (CX, CMZ, CAZ, CIP), aminoglycosides (GEN, K, PB, TOB), quinolones (MO, NX, NA, GAT), glycopeptides (TEI, VA) and methicillin (MET, β-lactams), with few exceptions. In contrast, high sensitivity was recorded against macrolides (AZM, E, CD), sulphonamides (COT, TR) and the carbapenems (IPM, MRP), with few variations. Varied susceptibility phenotypes were observed against the fusidanes and penicillins (Fig. 4). Percentage resistance to antibiotics was as follows: ≈ 100%, against ceftazidime, cefoxitin, kanamycin, nalidixic acid, vancomycin, teicoplanin, methicillin and norfloxacin; 91.2–97.2%, for cefmetazole, polymyxin B, tobramycin and moxifloxacin; and 76.5%, 76.5%, 79.4% and 82.4%, against ciprofloxacin, gentamycin, fusidic acid, and gartifloxacin, respectively. Intermediate resistance was observed against penicillin G and clindamycin (52.9 and 58.5%), while high sensitivity (70.6–100%) was recorded towards the remaining antibiotics: amoxiclav (Amoxicillin-clavulanate), tetracycline, tigecycline, meropenem, imipenem, trimethoprim, cotrimoxazole and azithromycin (intermediate values were all considered susceptible according to EFSA [10].

**Table 4:** Antibiotic susceptibility pattern of LAB isolates against different antibiotics recorded in terms of zone of inhibition by disc diffusion assay. Superscripts denote zones of inhibition (ZOI); S = susceptible; R = resistant; I = intermediate susceptible; AZM - Azithromycin; E - Erythromycin; CD - Clindamycin; CX - Cefoxitin; CMZ - Cefmetazole; CAZ - Ceftazidime; CIP - Ciprofloxacin; COT - Cotrimoxazole; TR - Trimethoprim; FC - Fusidane; GEN - Gentamycin; K - Kanamycin; PB - Polymyxin-B; TOB - Tobramycin; IPM - Imipenem; MRP- Meropenem; MET - Methicillin; MO - Moxifloxacin; NX - Norfloxacin; NA - Nalidixic acid; GAT - Gatifloxacin; P - Penicillin; AMC - Amoxiclav; TEI - Teicoplanin; VA - Vancomycin; TGC - Tigecycline; TE - Tetracycline

	Macrolides			Cephalosporins				Sulfonamides		Fusidane	Aminoglycosides				Beta-lactams			Quinolones				Penicilins		Glycopeptides		Tetracyclines	
	AZM	E	CD	CX	CMZ	CAZ	CIP	COT	TR	FC	GEN	K	PB	TOB	IPM	MRP	MET	MO	NX	NA	GAT	P	AMC	TEI	VA	TGC	TE
**GR4**	S^19^	S^19^	I^15^	R	R	R	I^15^	I^16^	S^18^	R	R^12^	R^0^	R^0^	R^11^	S^24^	S^18^	R^11^	R^11^	R	R	I^16^	R^14^	I^15^	R	R	S^27^	S^21^
**GR13**	R^11^	S^17^	I^15^	R	R	R	R^0^	S^20^	S^19^	R^13^	R^11^	R^0^	R^0^	R^0^	S^32^	I^16^	R^0^	R^0^	R	R	R^0^	S^20^	S^20^	R	R	S^20^	I^16^
**GR8**	I^15^	S^20^	I^15^	R	R	R	R^11^	S^17^	S^17^	R	R^12^	R^0^	R^0^	R^0^	S^21^	R^13^	R^0^	R^12^	R	R	R^11^	R^11^	S^17^	R	R	S^17^	R^12^
**GR27**	S^20^	S^24^	R	R	R^0^	R	S^21^	I^16^	S^18^	R	S^22^	R^12^	S^17^	R^13^	S^22^	S^26^	R^13^	R^0^	R	R	S^22^	I^15^	S^20^	R	R	S^36^	S^21^
**GR11**	R^13^	I^16^	R^12^	R	R^10^	R	R^10^	R^10^	S^17^	I^15^	R^11^	R	R	R	S^30^	R^13^	R	I^15^	R^14^	R^12^	R^0^	I^15^	S^21^	R	R	S^17^	R
**GR5**	I^16^	S^21^	S^24^	R^11^	R	R	R	S^17^	S^20^	R	I^15^	R	R	R	S^27^	I^15^	R	R^12^	R	R	R^12^	R	S^21^	R	R	S^17^	I^15^
**GR2**	R^12^	S^19^	R	R	R	R	R	S^20^	I^16^	R^11^	S^18^	R	R^13^	R^0^	S^22^	I^16^	R	R	R	R	S^18^	R^12^	S^20^	R	R	S^20^	S^20^
**GR29**	S^18^	S^22^	R	R	R	R	R^10^	S^17^	S^21^	R	S^19^	R	R^14^	R^10^	S^23^	I^16^	R^10^	R^12^	R	R^11^	R^0^	S^20^	S^21^	R	R	S^20^	S^17^
**GR32**	S^17^	S^20^	R	R	R	R	I^16^	S^20^	S^22^	R	R^12^	R	R	R^0^	S^23^	S^21^	R	R	R^12^	R	R^12^	R	R^12^	R	R	S^27^	S^21^
**GR12**	I^16^	S^23^	I^15^	R	R	R	R^12^	I^15^	S^22^	I^15^	R^14^	R	R	R^10^	S^27^	S^19^	R^10^	R^11^	R	R^10^	R^0^	S^20^	R^14^	R	R	S^21^	S^18^
**KN10**	I^15^	I^16^	R^13^	R	R	R	R	S^17^	S^17^	R	R^12^	R	R	R^0^	S^22^	S^17^	R	R	R	R	R^0^	I^15^	R^11^	R	R	S^18^	S^19^
**KN3**	I^15^	S^22^	R^12^	R	R	R	R^12^	I^15^	R	I^16^	R^11^	R	R	R^0^	S^36^	I^15^	R	R	R	R^10^	R^11^	S^23^	S^21^	R	R	S^22^	R^10^
**KN6**	I^15^	S^21^	R^12^	R	R	R	S^18^	S^19^	S^18^	R	R^11^	R	R	R^0^	S^24^	S^23^	R	R^12^	R^13^	R	S^17^	R^13^	S^20^	R	R	S^25^	S^21^
**KN5**	S^17^	S^22^	R^11^	R	R	R	R^10^	S^18^	S^20^	S^12^	R^12^	R	S^18^	R^0^	S^31^	I^15^	R	R^12^	R	R^11^	R^10^	R^13^	S^17^	R	R	S^15^	S^18^
	**AZM**	**E**	**CD**	**CX**	**CMZ**	**CAZ**	**CIP**	**COT**	**TR**	**FC**	**GEN**	**K**	**PB**	**TOB**	**IPM**	**MRP**	**MET**	**MO**	**NX**	**NA**	**GAT**	**P**	**AMC**	**TEI**	**VA**	**TGC**	**TE**
**KN9**	R^12^	S^21^	I^15^	R	R	R	R	R^13^	R^13^	S^19^	R^13^	R	I^16^	R^0^	S^32^	R^15^	R	R	R	R^11^	R	S^18^	I^15^	R	R	S^17^	R^13^
**BK5**	S^21^	S^25^	I^15^	R	R	R	I^16^	S^22^	S^24^	R^10^	S^18^	R	R	R^14^	S^24^	I^16^	R^14^	R	R	R	S^18^	S^19^	R^14^	R	R	S^21^	S^21^
**BK4**	I^15^	S^21^	R^11^	R	R	R	S^17^	I^16^	S^18^	R	R^10^	R	R	R^10^	S^21^	S^19^	R^10^	R^12^	R^10^	R	I^15^	S^18^	S^17^	R	R	S^22^	S^18^
**BK8**	S^18^	S^23^	R	R	R	R	R^10^	S^20^	S^24^	R^13^	R^12^	R	R^13^	R^0^	S^25^	I^16^	R	R^12^	R	R	R	I^15^	R^13^	R	R	S^17^	S^18^
**PT1**	S^20^	S^22^	S^22^	R^10^	R	R	R	I^15^	S^22^	R	R^13^	R	R	R^0^	S^22^	S^18^	R	R^12^	R^10^	R	R	R^14^	I^16^	R	R	S^21^	S^20^
**AK1**	S^18^	S^20^	R^14^	R	R	R	R^14^	S^18^	S^18^	R	R^14^	R	R10	R^0^	S^25^	S^19^	R	R^10^	R	R	R^12^	R^13^	R^13^	R	R	S^27^	R^13^
**AK5**	S^19^	S^21^	S20	R	R	R	R^10^	S^18^	S^22^	R^11^	R^12^	R	R	R^10^	S^24^	R^15^	R^10^	R^11^	R	R	R^10^	R	R^14^	R	R	S^19^	S^22^
**NON4**	S^22^	S^20^	R^12^	R	R	R	R^14^	S^21^	S^20^	R	I^16^	R	R^13^	R	S^23^	I^16^	R	R^11^	R	R	R^0^	R^10^	I^16^	R	R	S^20^	S^21^
	**AZM**	**E**	**CD**	**CX**	**CMZ**	**CAZ**	**CIP**	**COT**	**TR**	**FC**	**GEN**	**K**	**PB**	**TOB**	**IPM**	**MRP**	**MET**	**MO**	**NX**	**NA**	**GAT**	**P**	**AMC**	**TEI**	**VA**	**TGC**	**TE**
**3ST2**	S^17^	S^21^	I^15^	R	R	R	I^15^	R^14^	I^15^	I^16^	I^15^	R	R	R	S^25^	S^20^	R	R^12^	R	R	R^14^	R^13^	R^14^	R	R	S^24^	S^17^
**3ST3**	S^21^	S^25^	S^25^	R	R	R	R^14^	S^20^	S^19^	R^11^	R^13^	R	R	R	S^25^	S^19^	R	I^15^	R	R	R^13^	S^21^	S^21^	R^10^	R	I^16^	S^25^
**3ST5**	S^20^	S^25^	R^11^	R	R	R	R^14^	S^18^	S^22^	R^13^	R^12^	R	R	R	S^24^	S^19^	R	R^11^	R	R	R^14^	R	R^13^	R	R	S^23^	S^19^
**3ST7**	S^17^	S^20^	R	R	R	R	R	S^17^	R^0^	R	R^14^	R	R	R	S^22^	I^15^	R	R^0^	R	R	R^10^	R	R^13^	R	R	I^16^	S^20^
**3BM1**	R^13^	I^16^	R^14^	R	R	R	R	I^15^	S^21^	I^15^	R^11^	R	R^10^	R	S^31^	I^15^	R^10^	R^0^	R	R	R	R^13^	S^21^	R	R	S^22^	R^12^
**3BM3**	S^18^	S^23^	R^13^	R	R	R	I^15^	S^18^	S^17^	R	R^12^	R	R	R	S^23^	I^15^	R	R^0^	R	R	R^12^	R^14^	S^17^	R	R	S^21^	S^17^
**3BM4**	S^19^	S^23^	S^23^	R	R	R	R^12^	S^19^	S^22^	R^11^	R^13^	R	R	R	S^25^	I^15^	R	R^10^	R	R	I^15^	R^10^	I^15^	R	R	S^19^	S^24^
**8BM6**	S^19^	S^20^	S^19^	R	R	R	R	S^20^	S^19^	R	R^13^	R	R	R	S^24^	S^17^	R	R^11^	R	R	I^15^	I^16^	I^15^	R	R	S^20^	S^21^
**8BM9**	R^14^	S^19^	R	R	R	R	R	I^16^	S^18^	S^17^	R^13^	R	R^14^	R^14^	S^24^	I^15^	R	S^19^	R	R	R^10^	S^19^	S^18^	R	R	S^16^	I^15^
**8ST5**	S^19^	S^21^	R	R	R	R	R^12^	S^22^	S^22^	R	R^14^	R	R^13^	R^13^	S^25^	R^14^	R	R^12^	R	R	R	R	S^22^	R	R	S^20^	R^14^
**8ST7**	S^21^	S^24^	I^16^	R^10^	R	R	R	R^14^	S^17^	S^20^	S^18^	R	R	R	S^30^	R^14^	R	R^13^	R	R	R^12^	S^17^	S^26^	R	R	S^20^	I^15^
**15ST2**	R^14^	2^S0^	R^12^	R	R	R	R	S^19^	S^18^	R^11^	R^12^	R	R^14^	R^14^	S^26^	S^18^	R^13^	R^11^	R	R	R	S^17^	S^18^	R	R	S^25^	I^15^

**Fig. 5:**
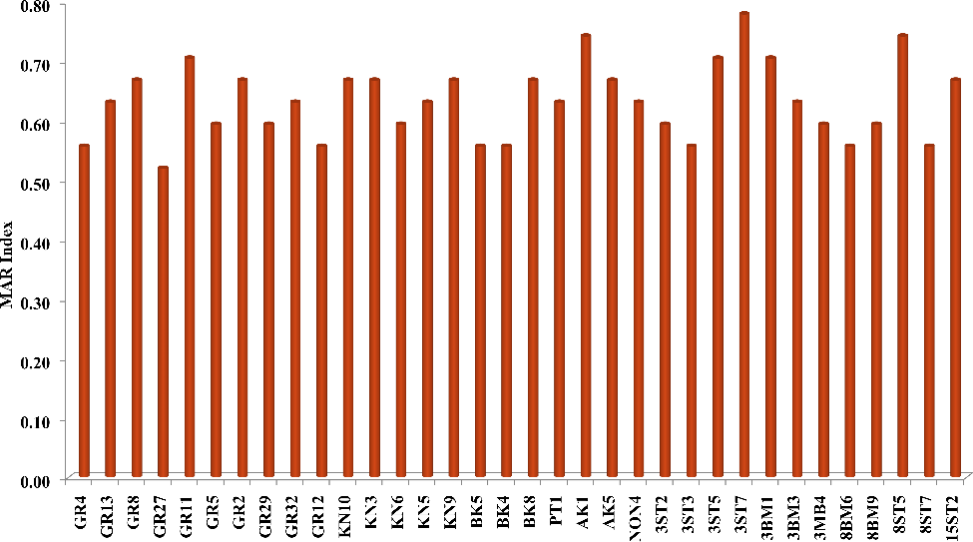
Distribution of multiple antibiotic resistance (MAR) index among lactic acid bacteria isolates

Isolates’ MAR indices (MAR), defined as resistance to up to 3 or more classes of antimicrobials, are presented in Fig. 5. MAR values of > 0.2 represented high risk sources of contamination (e.g., a source characterized by constant use of antibiotics). MAR indices were highest (MAR = 0.78, 0.74, respectively) with 3ST7 (*Lacticaseibacillus casei*) and 8ST7 (*Limosilactobacillus fermentum*), both the isolates from infant stool. These were followed by isolates AK1 (*Lacticaseibacillus casei*) from fermented akpu, and GR11 (*Lacticaseibacillus paracasei*) from garri (MAR = 0.74, and 0.70, respectively). The lowest MAR indices were obtained with GR27 (*Lacticaseibacillus casei*) from garri (MAR = 0.52), 3ST3 (*Lacticaseibacillus paracasei*) from infant faeces (0.56) and 8BM6 (*Lacticaseibacillus casei*) from human milk (0.56). Overall, each isolate exhibited multidrug resistance (MDR) towards the tested antibiotics with MAR index significantly higher than 0.2.

### Detection of antibiotic resistance genes

**Fig. 6:**
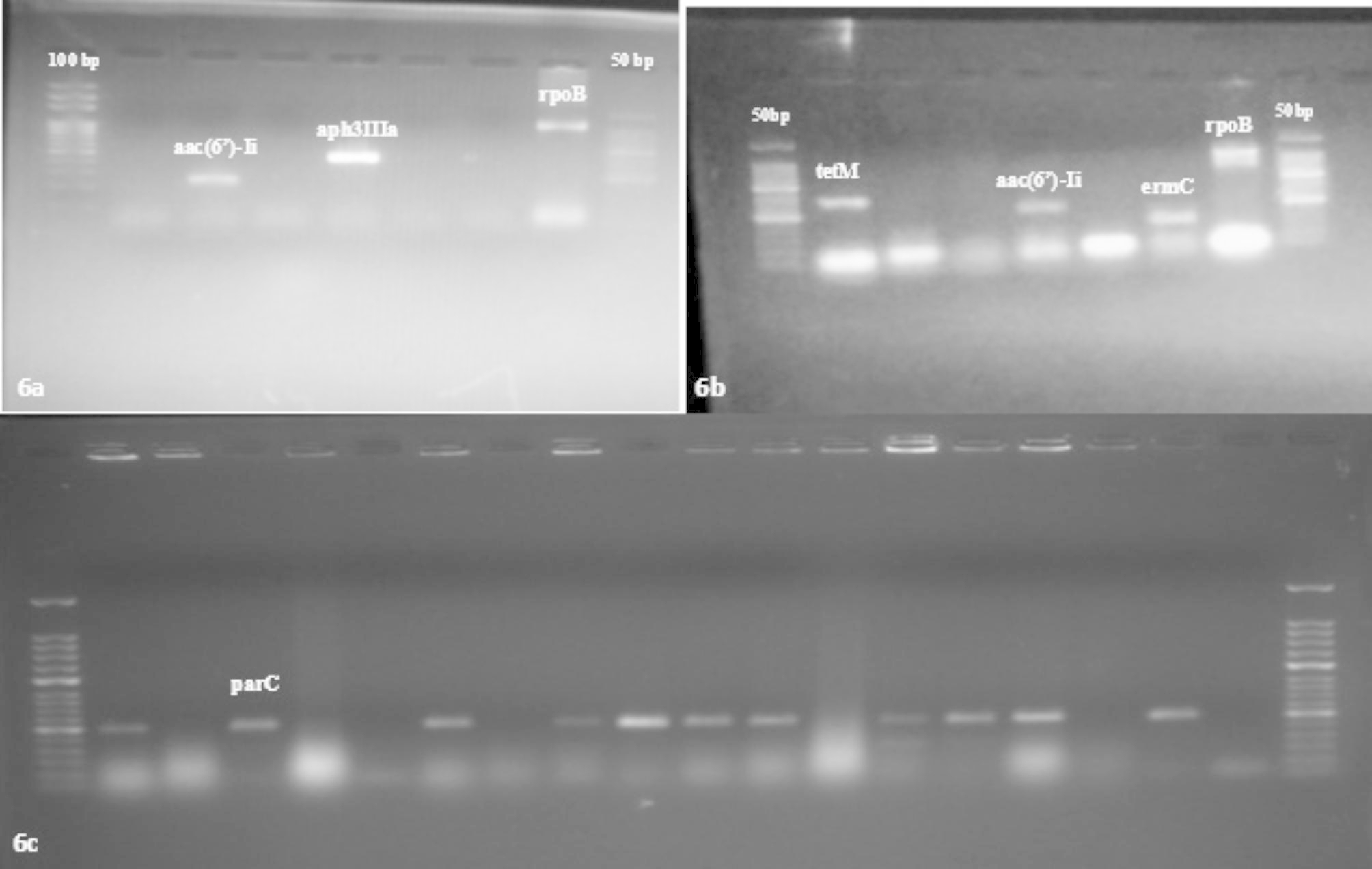
**a-c**: Gel documentation image showing amplified product for antibiotic resistance genes present in LAB isolates

LAB strains, displaying varied resistance phenotypes, were screened for presence of target antibiotic resistance coding genes (Table 2). Initial primer screening results revealed the presence of: *tetM* (406 bp, 80.0™); *aac(6’)-Ii* (410 bp, 83.0™); *ermB* (745 bp, 86.5™); *ermC* (299 bp, 85.5™); *aph3IIIa* (523 bp, 85.0™); *int-Tn* (1028 bp, 85.0™); *vanE* (513 bp, 80.0™), and *parC* (286 bp, 85.0™) (Fig. 6a-c**)**. No amplification was observed for *tetK*, *tetL*, *tetO*, *tetW*, *aph(2’)-Ia*, *blaZ*, *mecA*, *ant(4’)-Ia*, *ant(6’)-Ia*, *aph(2’’)-Ic*, *aph(2’’)-Id*, *aadE*, *dfrA*, and *lnuA*. Individual screening of isolates for genes encoding aminoglycoside resistance showed that 14 (41.2%) strains (GR4, GR27, GR2, GR32, GR12, KN6, BK4, BK8, PT1, AK5, 3ST2, 3ST5, 3BM4, 8BM6) contained chromosomally encoded *aac(6’)-Ii*; while *aph3IIIa* was detectable only in one (2.9%) strain (GR2). The other genes encoding aminoglycoside resistance (*aac(6’)-Ie-aph(2’’)-Ia*, *ant(4’)-Ia*, *ant(6’)-Ia*, *aadE*), *aph(2’’)-Id*, *aph(2’’)-Ic*) remained un-amplified.

Macrolide resistance-encoding genes, *ermC* and *ermB* were detected among 9 and 8 isolates, respectively. Both ARGs were detected in 5 isolates (GR2, GR32, KN6, 3ST2, and 8BM6), while only one of either was detectable in 7 isolates (*ermC*: PT1, AK1, BK4 and 8BM9; *ermB*: 3BM1, NON4, and 3ST5). All macrolide resistance-encoding genes (*ermA*, *mefA*/*E* and *lnuA*) were undetected. Only one tetracycline resistance gene (*tet(M)*) was detected among 7 strains (GR11, GR12, BK4, AK5, 3ST2, 3ST7, 8BM6). One isolate, 8BM6, showed presence of *int-Tn (Tn916-Tn1545*) transposable element. Penicillin resistance genes *blaZ*, *bla*, and *mecA* were absent throughout. Vancomycin resistance gene, *vanE* was detected among 16 (47.0%) isolates. Percentage occurrence of *parC* (resistance to ciprofloxacin) was 76.5%.

In several instances, phenotype-genotype correlation could not be established. Strains 3ST2, 3ST7, 8BM6, GR12, BK4, and AK5 were susceptible to tetracycline, despite harbouring chromosomally encoded *tetM* gene. Also, all strains were vancomycin-resistant, despite only 47.0% of strains were positive for *vanE* (Table 5**)**. ARGs were absent in six strains, 3BM3, 8ST7, GR8, KN5, KN10, BK5.

**Table 5:** Phenotypic-genotypic correlation of antibiotic resistance in LAB isolates from human and Nigerian fermented food sources

Isolates	Antibiotics Resistance Phenotype	Resistance genes detected by PCR
*Lacticaseibacillus casei* 3ST2	CX, CMZ, CAZ; COT, K, FC, TOB; MET; MO, NX, NA, GAT; P, AMC; TEI, VA	*aac(6’)-Ii*, *ermB*, *ermC*, *tetM*, *vanE*, *parC*
*Lacticaseibacillus casei* 3ST5	CD; CX, CMZ, CAZ; CIP, FC, GEN, K, PB, TOB, MET, MO, NX, NA, GAT, P, AMC, TEI, VA	*aac(6’)-Ii*, *ermB*, *parC*
*Lacticaseibacillus casei* 3ST7	CD, CX, CMZ, CAZ, CIP, TR, FC, GEN, K, PB, TOB, MET, MO, NX, NA, GAT, P, AMC, TEI, VA	*tetM*
*Lacticaseibacillus paracasei* 3ST3	CX, CMZ, CAZ, CIP, FC, GEN, K, PB, TOB, MET, NX, NA, GAT, TEI, VA	*parC*
*Lactiplantibacillus pentosus* 8ST5	CD, CX, CMZ, CAZ, CIP, FC, GEN, K, PB, TOB, MRP, MET, MO, NX, NA, GAT, P, TEI, VA, TE	*vanE*, *parC*
*Lactiplantibacillus plantarum* 15ST2	AZM, CD, CX, CMZ, CAZ, CIP, FC, GEN, K, PB, TOB, MET, MO, NX, NA, GAT, TEI, VA	*parC*
*Lacticaseibacillus paracasei* 3BM4	CX, CMZ, CAZ, CIP, FC, GEN, K, PB, TOB, MET, MO, NX, NA, P, TEI, VA	*aac(6’)-Ii*, *vanE*, *parC*
*Lactiplantibacillus plantarum* 3BM1	AZM, CD, CX, CMZ, CAZ, CIP, GEN, R, PB, TOB, MET, MO, NX, NA, GAT, P, TEI, VA, TE	*ermB*, *ermC*, *vanE*, *parC*
*Lacticaseibacillus casei* 8BM6	CX, CMZ, CAZ, CIP, FC, GEN, K, PB, TOB, MET, MO, NX, NA, TEI, VA	*aac(6’)-Ii*, *ermB*, ermC, *int-Tn(Tn916)-Tn1545*, *tetM*, *vanE*, *parC*
*Levilactobacillus brevis* 8BM9	AZM, CD, CX, CMZ, CAZ, CIP, GEN, K, PB, TOB, MET, NX, NA, GAT, TEI, VA	*ermC*, *tetM*, *vanE*, *parC*
*Lacticaseibacillus casei* GR4	CX, CMZ, CAZ, CIP, FC, GEN, K, PB, TOB, MET, MO, NX, NA, P, TEI, VA	*aac(6’)-Ii*, *vanE*, *parC*
*Levilactobacillus brevis* GR2	AZM, CD, CX, CMZ, CAZ, CIP, FC, K, PB, TOB, MET, MO, NX, NA, P, TEI, VA	*aac(6’)-Ii*, *ermB*, *ermC*, *aph3IIIa*, *parC*
*Levilactobacillus brevis* GR5	CX, CMZ, CAZ, CIP, FC, K, PB, TOB, MET, MO, NX, NA, GAT, P, TEI, VA	*parC*
*Lacticaseibacillus paracasei* GR11	AZM, CD, CX, CMZ, CAZ, CIP, COT, GEN, K, PB, TOB, MRP, MET, NX, NA, GAT, TEI, VA, TE	*tetM*, *vanE*, *parC*
*Levilactobacillus brevis* GR29	CD, CX, CMZ, CAZ, CIP, FC, K, PB, TOB, MET, MO, NX, NA, GAT, TEI, VA	*vanE*, *parC*
*Lacticaseibacillus casei* GR27	CD, CX, CMZ, CAZ, FC, K, TOB, MET, MO, NX, NA, TEI, VA	*aac(6’)-Ii*, *vanE*, *parC*
*Lacticaseibacillus casei* GR32	CD, CX, CMZ, CAZ, FC, GEN, K, PB, TOB, MET, MO, NX, NA, GAT, P, AMC, TEI, VA	*aac(6’)-Ii*, *ermB*, *ermC*, *parC*
*Lacticaseibacillus paracasei* GR12	CX, CMZ, CAZ, CIP, GEN, K, PB, TOB, MO, NX, NA, GAT, AMC, TEI, VA	*aac(6’)-Ii*, *tetM*, *vanE*, *parC*
*Levilactobacillus brevis* GR13	AZM, CX, CMZ, CAZ, CIP, FC, GEN, K, PB, TOB, MET, MO, NX, NA, GAT, TEI, VA	*parC*
*Lacticaseibacillus casei* KN3	CD, CX, CMZ, CAZ, CIP, TR, GEN, K, PB, TOB, MET, MO, NX, NA, GAT, TEI, VA, TE	*vanE*
*Levilactobacillus brevis* KN9	AZM, CX, CMZ, CAZ, CIP, COT, TR, GEN, K, TOB, MRP, MET, MO, NX, NA, GAT, TEI, VA, TE	*vanE*
*Lacticaseibacillus casei* KN6	CD, CX, CMZ, CAZ, FC, GEN, K, PB, TOB, MET, MO, NX, NA, P, TEI, VA	*aac(6’)-Ii*, *ermB*, *ermC*, *parC*
*Lacticaseibacillus paracasei* BK4	CD, CX, CMZ, CAZ, FC, GEN, K, PB, TOB, MET, MO, NX, NA, TEI, VA	*aac(6’)-Ii*, *ermC*, *tetM*, *vanE*, *parC*
*Levilactobacillus brevis* BK8	CD, CX, CMZ, CAZ, CIP, FC, GEN, K, PB, TOB, MET, MO, NX, NA, GAT, AMC, TEI, VA	*aac(6’)-Ii*, *vanE*
*Lacticaseibacillus paracasei* PT1	CX, CMZ, CAZ, CIP, FC, GEN, K, PB, TOB, MET, MO, NX, NA, GAT, P, TEI, VA	*aac(6’)-Ii*, *ermC*, *vanE*
*Lacticaseibacillus casei* AK1	CD, CX, CMZ, CAZ, CIP, FC, GEN, K, PB, TOB, MRP, MET, MO, NX, NA, GAT, P, AMC, TEI, VA, TE	*vanE*, *parC*
*Lacticaseibacillus casei* AK5	CX, CMZ, CAZ, CIP, FC, GEN, K, PB, TOB, MRP, MET, MO, NX, NA, GAT, P, AMC, TEI, VA	*aac(6’)-Ii*, *tetM*, *vanE*, *parC*
*Lacticaseibacillus paracasei* NON4	CD, CX, CMZ, CAZ, CIP, FC, K, PB, TOB, MET, MO, NX, NA, GAT, P, AMC, TEI, VA	*ermB*, *parC*

## Discussion

Lactobacilli constitute a highly diverse and heterogeneous group of bacteria. Clustering of MALDI-TOF mass spectra retrieved for each isolate with those of taxonomically well characterised reference strains in a reference database and the BioNumerics software [39], allowed identification of our isolates to species levels. Correlation of MALDI-TOF MS identification to 16 S rRNA gene sequencing results showed 66.7% concurrence, as few isolates revealed different species-level identities with 16 S rRNA sequencing. Few recent reports have also documented similar identity dichotomy among lactobacilli identified using both methods. The negative concordance between MALDI-ToF MS and 16 S identification techniques may be due to difficulty of obtaining satisfactory spectra from some species and partly due to the limit of spectra in commercial reference libraries [40–42]. Kim et al. (2022b) [42] suggested analyzing spectra for species-specific protein peaks for overcoming the database limitations. They successfully employed the same for differentiation and identification of *L. casei*, *L. paracasei*, *L. rhamnosus*, *L. chiayiensis*, and *L. zeae.*

An important long-term objective of the present study is the application and establishment of indigenous lactobacilli of Nigerian origin as probiotics, for formulation of healthy and functional foods. As such, the harbouring of transferable antibiotic resistance coding genes, especially those of significant clinical importance, should constitute highly undesirable characteristics [43, 44]. Antibiotic resistance is a natural survival strategy exhibited by all microorganisms, including the LABs [45]. Two potential (a beneficial and a negative) outcomes have been associated with antibiotic resistance in probiotic microbes, depending on whether such antibiotic resistance is primary (intrinsic) or secondary (acquired). Intrinsic antibiotic resistance, because it is non-transferable, is considered an advantage in a probiotic bacterium, especially if the latter is to be co-administered with antibiotics (e.g., treatment of peptic ulcer due to *Helicobacter pylori* infection [46]; as it assures gut survival of the probiotic bacteria and prevents otherwise depletion of the gut’s natural microbiome [9]. Secondary antibiotics resistance is, on the other hand, of great clinical concern and not considered a good attribute for potential probiotic and starter LAB, given that the genes encoding antibiotic resistance may be transferred to potentially pathogenic organisms in vivo [47, 48].

Isolates displayed clear resistance to 10 antibiotics, including well-known inhibitors of cell wall synthesis (cefoxitin, cefmetazole, ceftazidime, methicillin, teicoplanin and vancomycin), protein synthesis (kanamycin and tobramycin), and nucleic acid synthesis (norfloxacin and nalidixic acid). Additionally, high-level resistance phenotypes were observed with polymyxin B and moxifloxacin (91%, respectively); while resistance towards gentamycin, fusidane and ciprofloxacin was moderately high (76.5%, respectively).

Overwhelmingly high resistance of isolates to the quinolones (100% for nalidixic acid and norfloxacin, and 94.1, 79.4, and 76.5% for moxifloxacin, gatifloxacin, and ciprofloxacin, respectively), is in concurrence to earlier report by Sharma et al. [25], who too observed similarly high resistance to the quinolones (≈ 83% to nalidixic acid) in their LAB isolates. High resistance to quinolones may be due to some [49, 50] intrinsic resistance mechanisms. This probably partially explains the high incidences of resistance towards norfloxacin, ciprofloxacin, moxifloxacin, nalidixic acid and gatifloxacin in the present study. Observations have been made that antibiotic susceptibility profile of lactobacilli may vary based on the isolation source [51]. In the current study, all incidences of sensitivity to quinolone antibiotics occurred only when the isolate was from a fermented food source, while isolates from human sources (stool or breast milk) were intermediate to predominantly resistant. It may, therefore, as was observed by others [51], appear that lactobacilli from food sources are more susceptible to ciprofloxacin and gatifloxacin than those from human sources. Higher incidences of quinolone resistance among human isolates may be attributed to the higher probability of exposure to antibiotics in their natural habitat (due to use/overuse in treatment and prophylaxis of bacterial infections) than are isolates from fermented foods [26, 52 ]. Most notably, *Lacticaseibacillus paracasei* NON4, the only isolate from fermented milk (fura de nono) was completely resistant to all 5 quinolone antibiotics. On-farm practices of supplementing animal feed with antibiotics greatly increase the propensities that LAB isolates from animal sources (including milk) could exhibit wide-ranging phenotypic antibiotic tolerances [53, 54].

High-level incidence of resistance among isolates to vancomycin and teicoplanin, strongly conforms to findings by Wang et al. [9]. Lactobacilli have high natural resistance to glycopeptide antibiotics, this character being engendered by chromosomally encoded differences in their peptidoglycan assembly pathway, which dictate substitution of regular microbial d-Ala-d-Ala dipeptide residues in the muramyl pentapeptide cell wall with d-Ala-d-lactate (high-level resistance) or d-Ala-d-Ser (low-level resistance) [9, 55].

In this study,, resistance towards penicillin G and ampicillin varied based on species with much higher resistance among *Lacticaseibacillus casei* (91.7%), than among *L. brevis* (45.5%) and *L. paracasei* (33.3%) strains. Similar trend was observed with amoxiclav. Reports of high-level resistance to penicillin antibiotics have also been made for *L. paracasei*, *L. casei*, *L. brevis*, and *L. plantarum* strains [9, 56]. Most notably, Olukoya et al. [57] reported high penicillin G resistance among lactobacilli of fermented food origin from Nigeria. Lactobacilli susceptibility to β-lactam antibiotics is reportedly higher towards the penicillins, but lower against cephalosporins [58]. All the isolates in this study displayed resistance against tested cephalosporins (cefoxitin, cefmetazole and ceftazidime), supporting the above view. Earlier, Osaro-Matthews and Nweke [59] observed marked (100%) resistance to cefotaxime by Nigerian LAB isolates. Although the physiological basis of the lactobacilli resistance to cephalosporins remain unclear, general processes, such as natural presence of broad-spectrum β-lactamases [60] and/or non-specific multidrug transporters/cell wall impermeability, have been proposed as possible explanations [61]. Impermeability associated with a defective cell wall-associated autolytic system, as was described for *Lacticaseibacillus casei* and *Lactiplantibacillus plantarum* [62, 63], could also possibly mediate natural cephalosporin resistance among lactobacilli. Reports have been made of the isolation of carbapenem-resistant lactobacilli [26]. Imipenem was one of the most effective of the antibiotics used in this study, inhibiting all the isolates. Conversely, some isolates showed resistance to meropenem. Species-related patterns in meropenem resistance were observed, with *Levilactobacillus brevis* strains giving 91.9% (outright resistant + intermediate resistant); while *Lactiplantibacillus pentosus* and *Lacticaseibacillus paracasei* gave 66.7 and 50%, respectively, compared to *Lacticaseibacillus casei* (38.5%) and *Lactiplantibacillus plantarum* (0%). These results are consistent with others’ observations [26, 64], but contradict Sharma and Goyal [65] who reported high-level sensitivity to meropenem. Felten [66] proposed that antibiotic susceptibility among lactobacilli could be species-specific. This seems to support the differential responses among lactobacilli species screened in this study.

Drago et al. [67] had reported high-level resistance to macrolides among lactobacilli strains, with one-third of their isolates showing resistance to macrolides, even prior to in vitro exposure to erythromycin. Such level of macrolide resistance was not observed in the current study. Instead, susceptibility to the tested macrolides was high, with 91.2 and > 79% of the strains being erythromycin- and azithromycin-sensitive, respectively, thus agreeing to observations by Danielsen and Wind [51] and Delgado et al. [61]. Lactobacilli sensitivity to macrolide antibiotics has been described as strain-dependent [67]. At 19 and 57% (fermented food) and 23 and 53% (human sources), respectively, resistance to azithromycin and clindamycin by isolates appeared not to be markedly influenced by their source of isolation.

In apparent agreement with others [26], very little or no inhibition of microbial growth by aminoglycosides was documented in the current work; especially against kanamycin and tobramycin. Gentamicin was however mildly effective, with about 14.7% of isolates showing sensitivity. Sharma et al. [26] and Adimpong et al. [68] have similarly observed gentamicin effectiveness against few isolates, but widespread and high-level resistance towards tobramycin and streptomycin by lactobacilli from milk and African fermented foods, respectively. Lactobacilli have been mostly resistant to aminoglycosides [69, 70], while the aminoglycoside resistance phenotype has been adjudged intrinsic; being mainly ascribed to two key factors: bacterial cell surface’ low permeability to aminoglycosides, and the absence, in lactobacilli, of elements of cytochrome-mediated electron transport [8].

Tetracycline, is among the most commonly used antibiotics in clinical therapy, and as growth promoters in animal husbandry and veterinary practice [71]. As a result, the potential for high incidences in resistance to the tetracyclines among microbial food cultures, and the likelihood that these may potentially become vehicles for onward spread of associated resistance genes to human pathogens should, naturally, be of great health concern. Our lactobacilli displayed susceptibility to tetracyclines; in concurrence to earlier reports showing active inhibition of lactobacilli by tetracyclines [26, 51]. The only two isolates not outright susceptible to tigecycline (*Lactocaseibacillus casei* 3ST7 and *Lacticaseibacillus paracasei* 3ST3), and showing intermediate resistant phenotypes, were from human sources (infant stool), suggesting a possible role for the source of lactobacilli in the frequency of their resistance to tetracyclines. Indeed, three (3) out of seven (7) of the stool isolates (or 42.9%) had a resistant or intermediate resistant phenotype to tetracycline in this study, compared to 25% and 31.8%, respectively, for breastmilk and fermented food isolates. Previously, Fontana et al. [71] had shown the importance of source as factor contributing to the incidence of tetracycline tolerance in lactobacilli. Chances that lactobacilli will encounter antibiotic drugs are higher for the human gut than they are for breastmilk or fermented food; probably explaining the much higher incidence of tetracycline resistance in stool isolates.

Isolates displaying resistance to trimethoprim and co-trimoxazole have quite often been reported [26, 51]. This was substantially contradicted by our observations, with majority of our isolates (85.3 and 64.7%, respectively) showing sensitivity to trimethoprim and co-trimoxazole (1:5 trimethoprim: sulfamethoxazole). Resistance to inhibitors of nucleic acid synthesis like trimethoprim and sulphonamides (e.g., co-trimoxazole) has been reported to be generally intrinsic in lactobacilli [72]. Sulphonamides and trimethoprim act by blocking the bacterial dihydrofolate reductase and dihydropteroid acid synthetase activities, respectively. Lactobacilli with natural resistance to sulphonamides and trimethoprim lack the folic acid biosynthetic pathway [73, 74]. Overwhelming susceptibility to both trimethoprim and co-trimoxazole by our isolates therefore suggests that these are biosynthetically capable of folic acid formation. Trimethoprim and sulfamethoxazole act at different steps during tetrahydrofolate formation; but, together, exert a synergistic action when combined (co-trimoxazole), implying higher effectiveness than trimethoprim. Most contrarily, our isolates were more susceptible to trimethoprim than to cotrimoxazole. We are immediately unable to provide clear explanations for above observation. The following factors previously highlighted [75] could, however, have played some role, viz.: differential cell wall impermeability towards trimethoprim and sulfamethoxazole; presence of alternative metabolic pathways; and existence of sulfamethoxazole-insensitive dihydrofolate reductase or its overproduction. It is additionally possible that determination of phenotypic susceptibility to co-trimoxazole, in some of our isolates, be influenced by antagonistic medium components, as has been shown for *p*-aminobenzoic acid (PABA) and thymidine by Turnidge and Bell [76]. Any combination of the above factors could have affected the lactobacilli inhibitory effectiveness of co-trimoxazole, compared to trimethoprim. This is especially so when it is considered that trimethoprim is a minor component (only 1/6 parts) of co-trimoxazole. Lactobacilli have been reported to be, naturally, highly resistant to fusidic acid [51, 77]. This was substantially borne out, in this study, as 76.4% fusidic acid resistance was observed. In agreement with Danielsen and Wind [51], susceptibility to fusidic acid appeared to be, somewhat, species-dependent, with 75% of all susceptible isolates being of *Levilactobacillus brevis*, and all *Lacticaseibacillus paracasei* isolates being resistant, also, in strong support of observations by Klare et al. [30].

Many reports documents the presence of antibiotic resistance genes in probiotic, as well as human- and food-associated strains of lactobacilli [7, 8, 77–80]. After phenotypic resistance to antibiotics is observed, it is necessary to identify the molecular basis of such resistance; especially in human- and food-associated microbial strains, to establish the possible transmissibility of such resistance. Assessment of antibiotic resistance at the genomic level returned marked antimicrobial gene diversity, although only 8 genes (*tetM* (tetracycline resistance); *ermB* and *ermC* (erythromycin resistance); *vanE* (vancomycin resistance); *parC* (quinolone resistance); *aac(6’)-Ii* and *aph3IIIa* (aminoglycoside resistance), with the conjugative transposable sequence element *int-Tn* (*Tn916/Tn1545*), were detected out of the ARGs assayed. Contrary to previous descriptions of the tetracycline (*tetM*) and erythromycin (*ermB*) resistance determinants, as the most commonly occurring antibiotic resistance genes among lactobacilli [8, 71] both genes, at individual incidences of 28.6%, were jointly 5th most detected resistance determinants in the tested strains, after *parC* (82.1%), *vanE* (64.3%), *aaC* (41.2%) and *ermC* (32.1%).

Three major mechanisms, efflux pumps, ribosomal protection proteins (RPPs), and direct enzymatic inactivation, account for tetracycline resistance among lactobacilli; while > 50 tetracycline resistance genes have, to date, been identified and characterized [81]. The detection of only *tetM*, one of three RPPs-coding genes, of the 5 tetracycline resistance determinants investigated, is strongly supported by research reporting *tetM* as predominant tetracycline resistance determinant among lactobacilli [8, 71, 81, 82]. On the other hand, non-detection of any efflux protein gene (*tetK* or *tetL*), while it agrees with others’ reports [81, 83], highlights the possible pre-eminence of RPPs-based mechanisms in lactobacilli resistance to tetracyclines. Reports have however been made of the identification of the tetracycline resistance-linked efflux pump-coding genes *tetK* and *tetL* in lactobacilli [84, 85]. Despite being identified in eight isolates, *tet(M)’s* presence coincided with the Tet^R^ phenotype in only one isolate, *Lacticaseibacillus paracasei* GR11, as all remaining *tetM*-bearing isolates, except the intermediately resistant *Lacticaseibacillus casei* 3ST7, and *Levilactobacillus brevis* 8BM9, were sensitive to tetracycline. Conversely, no ‘*tet’* genes was detected in the remaining five tetracycline-resistant isolates (8ST5, 3BM1, KN3, KN9, and AK1). Current observations of tetracycline resistance phenotype/genotype discrepancies in lactobacilli isolates are consistent with similar observations by others of tetracycline phenotypic/genotypic resistance dissonances in isolates from different origin [8, 83, 84]. Phenotype-genotype dissonance in tetracycline resistance, for our isolates, suggests possibl alternative mechanisms for tetracycline resistance. Others [8, 83], have proposed natural resistance or mutations [86]. It is additionally possible that *tetM* gene copies in these isolates simply failed to be expressed, due to some unknown factor. Also quite notable is the failure by Duskova et al. [8], to find any tetracycline resistance gene determinant in six tetracycline-resistant lactobacilli strains, despite using whole genome sequencing (WGS), and analysis with ResFinder and CARD. The relatively small number (5) of tetracycline resistance genes studied, compared to > 500 reported and characterized tetracycline resistance genes [84] could be another possible factor for the phenotype-genotype discrepancy.

The ‘*erm*’ genes, alongside ‘*tet*’ genes, have been proposed as most widespread lactobacilli ARGs associated with horizontal transfer [8]. Only *ermB* and *ermC*, of the macrolide resistance genes, were detected in our isolates, in agreement with Fontana et al. [71], although contrary to findings of those authors [71], correlation between *ermB* and *ermC* and phenotypic antibiotic resistance existed only for azithromycin-resistant *Levilactobacillus brevis* strains, but not for the erythromycin-resistant phenotype. Also, all tested strains of *Lacticaseibacillus casei* and *Lacticaseibacillus paracasei* bearing *ermB* and/or *ermC* were phenotypically sensitive to both macrolide antibiotics, except *Lacticaseibacillus paracasei* BK4, which showed intermediate sensitivity to azithromycin. Phenotypic resistance to the lincosamide clindamycin also correlated with *ermB* and/or *ermC* genotype, irrespective of the lactobacilli species; although several strains showing absence of corresponding resistance genes were clindamycin-resistant. Others [87] had also made similar observations on the genotype-phenotype association for their respective macrolide-resistant isolates. Identification only of the *erm* genes, in this study, suggests ribosome methylation being possibly the major mechanism of macrolide resistance, in agreement with others [71]. Regular reports have been made of genetic linkage of *erm* genes, especially *ermB*, and *tetM* [88, 89]. Co-occurrence of *tetM* and *ermB* and/or *ermC* genes was observed only for 4 isolates [3ST2, 8BM6, 8BM9 and BK4]. The possibility for genetic linkage can, at this time, be proposed only for *Lacticaseibacillus casei* 8BM6, the only isolate with detectable presence of a *Tn916/Tn1545* family transposon. *Tn916/Tn1545* family mobile genetic elements are well-known regular carriers of *tetM* and *ermB* genes [81]. *Tn916/Tn1545* transposon family members are both highly infective and capable of ready transfer to a wide variety of Gram-positive and Gram-negative bacteria [88, 89]. Detection in *Lacticaseibacillus casei* 8BM6 of *Tn916/Tn1545* markedly heightens chances for the horizontal transmission of associated resistance genes. It is notable that *Tn916/Tn1545* family also harbours other resistance determinants like the MAS (macrolide-aminoglycoside-streptothricin) element [88].

Mutations in the quinolone resistance-determining regions (QRDR) of the bacterial DNA gyrase and DNA topoisomerase IV genes (both genes encoding for enzymes essential for bacterial reproduction and transcription) form the genetic bases for lactobacilli resistance to fluoroquinolones [49]. The occurrence of the *parC* gene for fluoroquinolone resistance was, at 82.1% for the tested strains, the highest observed in this study, and probably also accounts to a large extent for the overwhelming levels of quinolone resistance observed in the current study; although *parC* amplification products were not, in this work, investigated for mutations. We cannot therefore state the extent to which resistance to quinolones by our isolates was due to the *parC* gene. Other factors possibly contributing to quinolone resistance could be cell wall impermeability [34, 84, 90] and mutations in the *gyrA* gene, previously associated with high-level quinolone resistance in lactobacilli [49, 50, 52]. Vancomycin resistance determinant, *vanE*, at 64.3% incidence among tested strains, was the second most detected ARG, despite absence of vancomycin resistance determinants in a large number of strains phenotypically resistant to teicoplanin and vancomycin. Several glycopeptide resistance-encoding genes have been identified in lactic acid bacteria, each associated with different ligase, engendering a wide spectrum of resistances to glycopeptides. Being largely chromosomally encoded, *vanE* is largely considered not to be horizontally transferable [67, 91, 92]. Lactobacilli glycopeptide antibiotic resistance has also been ascribed to alternative resistance mechanisms [73, 93], which probably explains the observed resistance in cells which lacked *vanE*. Only one vancomycin resistance determinant was studied. We cannot therefore write off possible roles played by unstudied genes.

Resistance to aminoglycoside antibiotics is well considered intrinsic in lactobacilli, originating from the low cell membrane permeability [21, 74, 83]. Genes encoding aminoglycosides-modifying enzymes (thus for aminoglycosides resistance) have, nevertheless, been reported in lactobacilli [8, 50, 94]. The bifunctional gene encoding high-level kanamycin resistance and high-level gentamicin resistance, *aac(6’)Ie-aph(2”)la*, has previously been reported [94, 95] but not detected, alongside four other aminoglycosides resistance determinant, *ant*, *aph(2’)-Ic, aph(2’)-Id* and *ant(6)-Ia*, in the current study. While *aac(6’)li* and *aph3IIIa* were detected, especially most concerning was the simultaneous occurrence, in *Lacticaseibacillus casei* 8BM6, of *aph3IIIa* and a *Tn916/Tn1545* transposon family member. In addition to *tetM* and *ermB*, the conjugative transmission of *aph3IIIa* has been associated with *Tn916/Tn1545* family elements [89, 96, 97]. Overwhelming resistance towards aminoglycosides by all our isolates, despite absence of corresponding resistance determinants in several, is ascribable to possible natural resistance [21, 62, 74, 83].

## Conclusion

The possibility that lactobacilli, in food fermentations and probiotic applications, may be sources for transferable antibiotic resistance genes to pathogens is legitimate cause of concern, for its implications for human health and food safety. Results demonstrate the occurrence of antibiotic resistance and presence of a selected pool of ARGs in 34 potential probiotic lactobacilli from Nigerian indigenous fermented foods and human sources. Only ARGs of the tested 25, were detectable with those conferring fluoroquinolone (parC) and glycopeptide (vanE) resistance occurring at the highest frequencies (82.1 and 64.3%, respectively). Most of the genes detected were chromosomal and, conceivably, pose low transmission risks, except for the highly concerning simultaneous detection, in one isolate, of *tetM*, *aac(6’)-li*, *ermB*, and *ermC* and a transposon of the highly infective Tn916/Tn1545 family. Sixteen isolates were multidrug resistant, while six isolates showed no evidence of ARGs. Little phenotype-genotype correlation in antibiotic resistance was observed throughout this study, except for very few instances, thus raising the possibility that resistance to antibiotics was probably mostly natural, although the above assertion may require further investigation to be confirmed. Overall, this study shows that lactobacilli from indigenous Nigerian fermented foods can contain antibiotic resistance genes to levels reported for other food matrices and may pose a similar health risk. Further research on the transmissibility of these AR genes, is required to confirm the safety of these isolates for probiotic and food fermentation applications.

## Data Availability

All data and materials shall be made available on request to Ms. Rachael T Duche. Email: duche20@gmail.com. For LAB DNA sequences, data has been deposited in ncbi.nlm.nih.gov with the following accession numbers: MF541063.1, MT515983.1, MT071603.1, MT613622.1, MN166310.1, KT589132.1, KJ726660.1, MN658813.1, MH704100.1, MH606197.1, MT613613.1, MT464356.1.

## Abbreviations

LAB: Lactic Acid Bacteria
GRAS: Generally Recognized As Safe
AMR: Antimicrobial Resistance
MDR: Multi-Drug Resistant
EFSA: European Food Safety Authority
QPS: Qualified Presumption of Safety
HM: Human Milk
RI: Reference Isolates
MALDI-TOF: Matrix Assisted Laser Desorption Ionization - Time of Flight
